# 3-(3-Chloro­benz­yl)-1*H*-isochromen-1-one

**DOI:** 10.1107/S1600536808030274

**Published:** 2008-09-27

**Authors:** Obaid-Ur-Rehman Abid, Ghulam Qadeer, Nasim Hasan Rama, Ales Ruzicka, Zdenka Padelkova

**Affiliations:** a Department of Chemistry, Quaid-I-Azam University, Islamabad 45320, Pakistan; b Department of General and Inorganic Chemistry, Faculty of Chemical Technology, University of Pardubice, Nam. Cs. Legii’ 565, 53210 Pardubice, Czech Republic

## Abstract

The asymmetric unit of the title compound, C_16_H_11_ClO_2_, a chemically synthesized isocoumarin, contains three independent mol­ecules. The benzopyran and benzene rings are approximately perpendicular to each other, forming dihedral angles ranging from 83.08 (14) to 87.43 (11)°. In the crystal structure, mol­ecules are linked by inter­molecular C—H⋯O hydrogen-bonding inter­actions, forming chains running parallel to the *a* axis.

## Related literature

For the properties and applications of isocumarins, see: Barry (1964 ▶); Powers *et al.* (2002 ▶); Sturtz *et al.* (2002 ▶). For the crystal structure of a related compound, see: Abid *et al.* (2006 ▶). For related literature, see: Allen *et al.* (1987 ▶); Rossi *et al.* (2003 ▶); Thomas & Jens (1999 ▶).
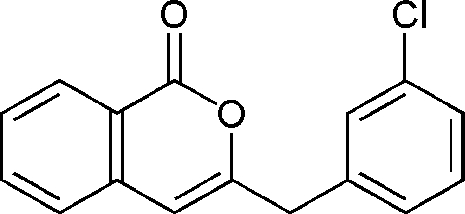


## Experimental

### 

#### Crystal data

C_16_H_11_ClO_2_
*M* *_r_* = 270.70
Triclinic, 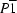

*a* = 8.1411 (8) Å
*b* = 15.0269 (14) Å
*c* = 16.4080 (16) Å
α = 91.696 (8)°
β = 98.478 (8)°
γ = 102.624 (6)°
*V* = 1933.4 (3) Å^3^
*Z* = 6
Mo *K*α radiation
μ = 0.29 mm^−1^
*T* = 150 (1) K
0.36 × 0.28 × 0.13 mm

#### Data collection

Bruker–Nonius KappaCCD area-detector diffractometer
Absorption correction: Gaussian (Coppens *et al.*, 1970 ▶) *T*_min_ = 0.930, *T*_max_ = 0.978
33806 measured reflections
8770 independent reflections
5387 reflections with *I* > 2σ(*I*)
*R*_int_ = 0.096

#### Refinement

*R*[*F* ^2^ > 2σ(*F* ^2^)] = 0.093
*wR*(*F* ^2^) = 0.280
*S* = 1.15
8770 reflections
514 parameters
H-atom parameters constrained
Δρ_max_ = 0.51 e Å^−3^
Δρ_min_ = −0.38 e Å^−3^

### 

Data collection: *COLLECT* (Hooft, 1998 ▶) and *DENZO* (Otwin­owski & Minor, 1997 ▶); cell refinement: *COLLECT* and *DENZO*; data reduction: *COLLECT* and *DENZO*; program(s) used to solve structure: *SIR92* (Altomare *et al.*, 1994 ▶); program(s) used to refine structure: *SHELXL97* (Sheldrick, 2008 ▶); molecular graphics: *PLATON* (Spek, 2003 ▶); software used to prepare material for publication: *SHELXL97*.

## Supplementary Material

Crystal structure: contains datablocks global, I. DOI: 10.1107/S1600536808030274/rz2245sup1.cif

Structure factors: contains datablocks I. DOI: 10.1107/S1600536808030274/rz2245Isup2.hkl

Additional supplementary materials:  crystallographic information; 3D view; checkCIF report

## Figures and Tables

**Table 1:** Hydrogen-bond geometry (Å, °)

*D*—H⋯*A*	*D*—H	H⋯*A*	*D*⋯*A*	*D*—H⋯*A*
C216—H216⋯O11	0.93	2.56	3.437 (6)	158
C116—H116⋯O21	0.93	2.58	3.396 (6)	147
C26—H26⋯O22^i^	0.93	2.45	3.339 (7)	161
C36—H36⋯O32^i^	0.93	2.52	3.409 (6)	160

Symmetry code: (i) 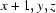.

**Fig. 1.**
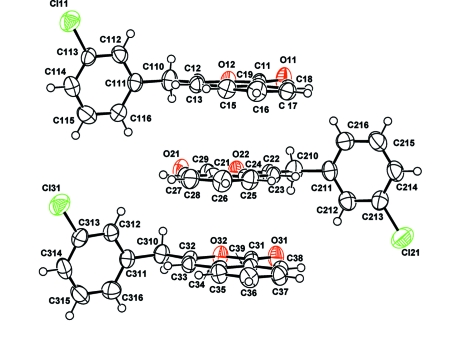
The asymmetric unit of the title compound, with displacement ellipsoids drawn at the 50% probability level.

**Fig. 2.**
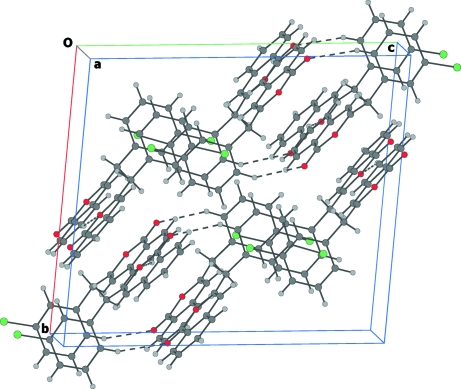
Crystal packing of the title compound viewed approximately along the a axis. Intermolecular C—H···O hydrogen bonds are shown as dashed lines.

**Fig. 3.**
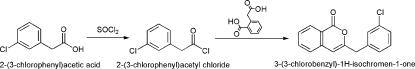
The formation of the title compound.

## supplementary crystallographic information

### Comment

The isocoumarin nucleus is an abundant structural motif in natural products (Barry, 1964). Many constituents of the steadily growing class of known isocoumarins exhibit valuable biological properties such as antifungal (Sturtz *et al.*, 2002), antitumor or cytotoxic, anti-inflammatory, anti-allergic (Rossi *et al.*, 2003) and enzyme inhibitory activity (Powers *et al.*,2002). Naturally occurring halo-isocoumarins and their halogeno-3,4-dihydroiscoumarin derivatives are very rare. However, a few examples of naturally occurring chlorine containing isocoumarins are known (Thomas & Jens, 1999). In view of the importance of this class of compounds, the title compound, an isocoumarine derivative containing a 3-chlorobenzyl substituent, has been synthesized and its crystal structure is reported here.

The asymmetric unit of the title compound contains three crystallographically independent molecules of similar geometry (Fig.1). The molecules are not planar, the dihedral angles formed by the benzopyran ring with the corresponding benzene ring being 83.08 (14), 87.43 (11) and 84.25 (14)° for the molecules containing Cl11, Cl21 and Cl31, respectively. The bond lengths (Allen *et al.*, 1987) and angles are within normal ranges and comparable with those reported for 3-(2-chlorobenzyl)isocoumarin (Abid *et al.*, 2006). In the crystal packing, molecules are linked by intermolecular C—H···O hydrogen bonds  (Table 1) into chains running parallel to the *a* axis (Fig. 2).

### Experimental

A mixture of 2-(3-chlorophenyl)acetic acid (4.76 g, 28 mmol) and thionyl chloride (2.94 ml, 34 mmol) was heated for 30 min in the presence of a few drops of DMF under reflux at 343 K to give 2-(3-chlorophenyl)acetyl chloride. Completion of the reaction was indicated by the disappearance of gas evolution. The removal of excess thionyl chloride was carried out under reduced pressure to afford 2-(3-chlorophenyl)acetyl chloride. Homophthalic acid (1.3 g, 7.2 mmol) was then added and the solution was refluxed for 6 hrs at 473 K with stirring. The reaction mixture was extracted with ethyl acetate (3 times 100 ml), and an aqueous solution of sodium carbonate (5%, 200 ml) was added to remove the unreacted homophthalic acid. The organic layer was separated, concentrated and chromatographed on silica gel using petroleum ether (313–353 K fractions) as eluent to afford the title compound (yield 62%; m.p. 350–351 K). Colourless single crystals suitable for X-ray analysis were obtained by slow evaporation of an ethyl acetate solution.

### Refinement

H atoms were positioned geometrically with C—H = 0.93–0.97Å and constrained to ride on their parent atoms, with *U*_iso_(H) = 1.2 *U*_eq_(C). The poor diffraction quality of the crystal may account for the high *R*_int_, weighted and unweighted *R* factors.

### Crystal data

**Table:**

C_16_H_11_ClO_2_	*Z* = 6
*M**_r_* = 270.70	*F*(000) = 840
Triclinic, *P*1	*D*_x_ = 1.395 Mg m^−^^3^
Hall symbol: -P 1	Melting point: 350(1) K
*a* = 8.1411 (8) Å	Mo *K*α radiation, λ = 0.71073 Å
*b* = 15.0269 (14) Å	Cell parameters from 33934 reflections
*c* = 16.4080 (16) Å	θ = 1–27.5°
α = 91.696 (8)°	µ = 0.29 mm^−^^1^
β = 98.478 (8)°	*T* = 150 K
γ = 102.624 (6)°	Block, colourless
*V* = 1933.4 (3) Å^3^	0.36 × 0.28 × 0.13 mm

### Data collection

**Table:**

Bruker–Nonius KappaCCD area-detector diffractometer	8770 independent reflections
Radiation source: fine-focus sealed tube	5387 reflections with *I* > 2σ(*I*)
graphite	*R*_int_ = 0.096
Detector resolution: 9.091 pixels mm^-1^	θ_max_ = 27.5°, θ_min_ = 1.8°
φ and ω scans to fill the Ewald sphere	*h* = −10→10
Absorption correction: integration (Gaussian; Coppens *et al.*, 1970)	*k* = −19→19
*T*_min_ = 0.930, *T*_max_ = 0.978	*l* = −21→21
33806 measured reflections	

### Refinement

**Table:**

Refinement on *F*^2^	Primary atom site location: structure-invariant direct methods
Least-squares matrix: full	Secondary atom site location: difference Fourier map
*R*[*F*^2^ > 2σ(*F*^2^)] = 0.093	Hydrogen site location: inferred from neighbouring sites
*wR*(*F*^2^) = 0.280	H-atom parameters constrained
*S* = 1.15	*w* = 1/[σ^2^(*F*_o_^2^) + (0.0832*P*)^2^ + 3.9759*P*] where *P* = (*F*_o_^2^ + 2*F*_c_^2^)/3
8770 reflections	(Δ/σ)_max_ < 0.001
514 parameters	Δρ_max_ = 0.51 e Å^−^^3^
0 restraints	Δρ_min_ = −0.38 e Å^−^^3^

### Special details

**Table:**

Geometry. All e.s.d.'s (except the e.s.d. in the dihedral angle between two l.s. planes) are estimated using the full covariance matrix. The cell e.s.d.'s are taken into account individually in the estimation of e.s.d.'s in distances, angles and torsion angles; correlations between e.s.d.'s in cell parameters are only used when they are defined by crystal symmetry. An approximate (isotropic) treatment of cell e.s.d.'s is used for estimating e.s.d.'s involving l.s. planes.
Refinement. Refinement of *F*^2^ against ALL reflections. The weighted *R*-factor *wR* and goodness of fit *S* are based on *F*^2^, conventional *R*-factors *R* are based on *F*, with *F* set to zero for negative *F*^2^. The threshold expression of *F*^2^ > σ(*F*^2^) is used only for calculating *R*-factors(gt) *etc*. and is not relevant to the choice of reflections for refinement. *R*-factors based on *F*^2^ are statistically about twice as large as those based on *F*, and *R*- factors based on ALL data will be even larger.

### Fractional atomic coordinates and isotropic or equivalent isotropic displacement parameters (Å^2^)

**Table:**

	*x*	*y*	*z*	*U*_iso_*/*U*_eq_	
Cl11	−0.0861 (2)	0.69111 (11)	0.78787 (9)	0.0721 (4)	
Cl31	−0.1878 (2)	0.34414 (12)	0.45567 (9)	0.0764 (5)	
Cl21	−0.2374 (3)	0.96729 (13)	−0.13645 (9)	0.0865 (6)	
O12	−0.3053 (4)	0.8902 (2)	0.4163 (2)	0.0466 (8)	
C34	−0.0819 (5)	0.5570 (3)	0.1009 (2)	0.0369 (9)	
O32	−0.4217 (4)	0.5627 (2)	0.09538 (19)	0.0452 (7)	
O22	−0.3792 (4)	0.7443 (2)	0.2320 (2)	0.0506 (8)	
C14	0.0400 (6)	0.8964 (3)	0.4355 (2)	0.0383 (9)	
C23	−0.1317 (6)	0.8252 (3)	0.1859 (3)	0.0401 (10)	
H23	−0.0798	0.8699	0.1540	0.048*	
C19	−0.0270 (6)	0.9565 (3)	0.3839 (3)	0.0386 (9)	
C11	−0.2077 (6)	0.9547 (3)	0.3750 (3)	0.0438 (10)	
O31	−0.4003 (4)	0.6731 (2)	0.0103 (2)	0.0534 (8)	
O11	−0.2803 (5)	1.0049 (3)	0.3363 (2)	0.0646 (10)	
C31	−0.3272 (6)	0.6231 (3)	0.0492 (3)	0.0394 (9)	
C29	−0.1117 (6)	0.7056 (3)	0.2811 (2)	0.0413 (10)	
C35	0.0900 (6)	0.5559 (3)	0.1035 (3)	0.0456 (11)	
H35	0.1377	0.5156	0.1362	0.055*	
C24	−0.0311 (6)	0.7732 (3)	0.2336 (3)	0.0391 (9)	
O21	−0.3788 (5)	0.6358 (3)	0.3182 (2)	0.0649 (10)	
C110	−0.3759 (6)	0.7710 (3)	0.5034 (3)	0.0513 (12)	
H110A	−0.4632	0.7392	0.4590	0.062*	
H110B	−0.4276	0.8088	0.5360	0.062*	
C32	−0.3542 (5)	0.5006 (3)	0.1420 (2)	0.0370 (9)	
C311	−0.4318 (5)	0.3711 (3)	0.2324 (3)	0.0416 (10)	
C13	−0.0723 (6)	0.8334 (3)	0.4773 (3)	0.0414 (10)	
H13	−0.0287	0.7937	0.5124	0.050*	
C22	−0.2965 (6)	0.8114 (3)	0.1858 (3)	0.0417 (10)	
C21	−0.2929 (6)	0.6906 (3)	0.2800 (3)	0.0471 (11)	
C113	−0.1807 (6)	0.6609 (3)	0.6859 (3)	0.0458 (11)	
C112	−0.2401 (6)	0.7239 (3)	0.6380 (3)	0.0476 (11)	
H112	−0.2317	0.7824	0.6608	0.057*	
C212	−0.3309 (6)	0.9170 (3)	0.0091 (3)	0.0516 (12)	
H212	−0.3752	0.8590	−0.0166	0.062*	
C12	−0.2366 (6)	0.8308 (3)	0.4666 (3)	0.0400 (10)	
C39	−0.1497 (5)	0.6193 (3)	0.0524 (2)	0.0349 (9)	
C18	0.0777 (7)	1.0171 (3)	0.3405 (3)	0.0488 (11)	
H18	0.0315	1.0559	0.3054	0.059*	
C310	−0.4898 (6)	0.4452 (3)	0.1844 (3)	0.0458 (11)	
H310A	−0.5882	0.4181	0.1435	0.055*	
H310B	−0.5251	0.4859	0.2219	0.055*	
C33	−0.1936 (6)	0.4969 (3)	0.1461 (3)	0.0402 (10)	
H33	−0.1513	0.4549	0.1787	0.048*	
C211	−0.3426 (6)	0.9338 (3)	0.0915 (3)	0.0472 (11)	
C17	0.2471 (7)	1.0200 (3)	0.3494 (3)	0.0548 (13)	
H17	0.3164	1.0606	0.3203	0.066*	
C38	−0.0470 (6)	0.6788 (3)	0.0070 (3)	0.0443 (10)	
H38	−0.0921	0.7204	−0.0250	0.053*	
C312	−0.3485 (6)	0.3892 (3)	0.3126 (3)	0.0443 (10)	
H312	−0.3297	0.4475	0.3380	0.053*	
C313	−0.2922 (6)	0.3210 (4)	0.3550 (3)	0.0505 (12)	
C111	−0.3126 (6)	0.7018 (3)	0.5568 (3)	0.0456 (11)	
C26	0.2337 (7)	0.7326 (4)	0.2817 (3)	0.0620 (14)	
H26	0.3502	0.7408	0.2815	0.074*	
C15	0.2150 (6)	0.9013 (4)	0.4447 (3)	0.0514 (12)	
H15	0.2634	0.8635	0.4800	0.062*	
C210	−0.4238 (6)	0.8580 (4)	0.1400 (3)	0.0551 (13)	
H210A	−0.4842	0.8822	0.1790	0.066*	
H210B	−0.5065	0.8132	0.1025	0.066*	
C214	−0.1859 (7)	1.0727 (3)	0.0021 (4)	0.0608 (14)	
H214	−0.1334	1.1192	−0.0279	0.073*	
C316	−0.4589 (7)	0.2834 (4)	0.1968 (3)	0.0588 (13)	
H316	−0.5154	0.2699	0.1429	0.071*	
C36	0.1877 (6)	0.6143 (3)	0.0579 (3)	0.0533 (12)	
H36	0.3019	0.6129	0.0597	0.064*	
C114	−0.1934 (9)	0.5750 (4)	0.6558 (4)	0.0702 (17)	
H114	−0.1541	0.5325	0.6889	0.084*	
C25	0.1436 (6)	0.7850 (4)	0.2350 (3)	0.0537 (12)	
H25	0.1998	0.8289	0.2038	0.064*	
C213	−0.2522 (7)	0.9871 (3)	−0.0338 (3)	0.0531 (12)	
C27	0.1542 (8)	0.6675 (4)	0.3290 (3)	0.0605 (14)	
H27	0.2174	0.6330	0.3612	0.073*	
C37	0.1202 (6)	0.6752 (3)	0.0099 (3)	0.0521 (12)	
H37	0.1887	0.7140	−0.0210	0.062*	
C315	−0.4023 (8)	0.2162 (3)	0.2408 (4)	0.0642 (15)	
H315	−0.4212	0.1575	0.2162	0.077*	
C216	−0.2756 (7)	1.0201 (4)	0.1280 (3)	0.0584 (13)	
H216	−0.2832	1.0321	0.1831	0.070*	
C314	−0.3189 (7)	0.2345 (3)	0.3200 (4)	0.0571 (13)	
H314	−0.2811	0.1891	0.3497	0.068*	
C16	0.3164 (7)	0.9627 (4)	0.4017 (3)	0.0583 (13)	
H16	0.4327	0.9652	0.4078	0.070*	
C28	−0.0167 (7)	0.6535 (3)	0.3290 (3)	0.0540 (13)	
H28	−0.0702	0.6095	0.3610	0.065*	
C215	−0.1957 (9)	1.0890 (4)	0.0837 (4)	0.0730 (17)	
H215	−0.1498	1.1469	0.1093	0.088*	
C115	−0.2687 (11)	0.5514 (4)	0.5746 (4)	0.089 (2)	
H115	−0.2785	0.4924	0.5527	0.107*	
C116	−0.3259 (9)	0.6148 (4)	0.5254 (3)	0.0667 (16)	
H116	−0.3745	0.5980	0.4707	0.080*	

### Atomic displacement parameters (Å^2^)

**Table:**

	*U*^11^	*U*^22^	*U*^33^	*U*^12^	*U*^13^	*U*^23^
Cl11	0.0741 (10)	0.0797 (10)	0.0526 (8)	0.0050 (8)	−0.0034 (7)	0.0057 (7)
Cl31	0.0903 (11)	0.0920 (11)	0.0506 (8)	0.0367 (9)	−0.0020 (7)	0.0105 (7)
Cl21	0.1028 (13)	0.0933 (12)	0.0544 (8)	−0.0025 (10)	0.0208 (8)	0.0008 (8)
O12	0.0378 (17)	0.0530 (19)	0.0531 (18)	0.0140 (14)	0.0116 (14)	0.0182 (15)
C34	0.032 (2)	0.043 (2)	0.036 (2)	0.0082 (17)	0.0075 (17)	0.0017 (17)
O32	0.0337 (16)	0.0549 (19)	0.0517 (18)	0.0161 (14)	0.0098 (14)	0.0185 (15)
O22	0.0402 (18)	0.057 (2)	0.057 (2)	0.0092 (15)	0.0171 (15)	0.0186 (16)
C14	0.041 (2)	0.042 (2)	0.035 (2)	0.0130 (18)	0.0101 (18)	0.0014 (18)
C23	0.041 (2)	0.041 (2)	0.041 (2)	0.0087 (18)	0.0126 (19)	0.0096 (18)
C19	0.043 (2)	0.038 (2)	0.037 (2)	0.0101 (18)	0.0108 (18)	0.0015 (17)
C11	0.049 (3)	0.045 (3)	0.041 (2)	0.016 (2)	0.009 (2)	0.0130 (19)
O31	0.0475 (19)	0.057 (2)	0.061 (2)	0.0210 (16)	0.0082 (16)	0.0224 (16)
O11	0.061 (2)	0.073 (2)	0.072 (2)	0.029 (2)	0.0198 (19)	0.036 (2)
C31	0.044 (2)	0.039 (2)	0.038 (2)	0.0132 (19)	0.0074 (18)	0.0084 (18)
C29	0.053 (3)	0.039 (2)	0.031 (2)	0.0072 (19)	0.0074 (19)	0.0004 (17)
C35	0.035 (2)	0.047 (3)	0.058 (3)	0.0152 (19)	0.009 (2)	0.011 (2)
C24	0.042 (2)	0.036 (2)	0.040 (2)	0.0106 (18)	0.0081 (18)	0.0016 (18)
O21	0.067 (2)	0.063 (2)	0.063 (2)	−0.0004 (19)	0.0221 (19)	0.0241 (18)
C110	0.042 (3)	0.057 (3)	0.056 (3)	0.008 (2)	0.013 (2)	0.019 (2)
C32	0.036 (2)	0.041 (2)	0.033 (2)	0.0084 (18)	0.0047 (17)	0.0063 (17)
C311	0.032 (2)	0.045 (2)	0.047 (2)	0.0045 (18)	0.0101 (18)	0.011 (2)
C13	0.047 (3)	0.042 (2)	0.041 (2)	0.018 (2)	0.0117 (19)	0.0103 (19)
C22	0.048 (3)	0.039 (2)	0.038 (2)	0.0057 (19)	0.0154 (19)	0.0094 (18)
C21	0.056 (3)	0.041 (2)	0.043 (2)	0.003 (2)	0.012 (2)	0.006 (2)
C113	0.047 (3)	0.048 (3)	0.041 (2)	0.004 (2)	0.012 (2)	0.011 (2)
C112	0.049 (3)	0.037 (2)	0.055 (3)	0.002 (2)	0.011 (2)	0.004 (2)
C212	0.045 (3)	0.047 (3)	0.058 (3)	0.003 (2)	0.002 (2)	0.005 (2)
C12	0.040 (2)	0.039 (2)	0.043 (2)	0.0098 (18)	0.0086 (18)	0.0100 (18)
C39	0.033 (2)	0.036 (2)	0.034 (2)	0.0064 (16)	0.0062 (16)	0.0011 (16)
C18	0.055 (3)	0.042 (2)	0.050 (3)	0.007 (2)	0.015 (2)	0.008 (2)
C310	0.038 (2)	0.053 (3)	0.048 (3)	0.010 (2)	0.011 (2)	0.012 (2)
C33	0.044 (2)	0.040 (2)	0.040 (2)	0.0149 (19)	0.0073 (19)	0.0097 (18)
C211	0.040 (3)	0.046 (3)	0.057 (3)	0.012 (2)	0.006 (2)	0.015 (2)
C17	0.060 (3)	0.049 (3)	0.055 (3)	0.001 (2)	0.027 (2)	0.003 (2)
C38	0.047 (3)	0.043 (2)	0.044 (2)	0.009 (2)	0.011 (2)	0.0070 (19)
C312	0.044 (3)	0.043 (2)	0.048 (2)	0.0092 (19)	0.011 (2)	0.008 (2)
C313	0.050 (3)	0.060 (3)	0.045 (3)	0.014 (2)	0.016 (2)	0.012 (2)
C111	0.043 (3)	0.044 (2)	0.049 (3)	0.0022 (19)	0.014 (2)	0.011 (2)
C26	0.051 (3)	0.075 (4)	0.068 (3)	0.027 (3)	0.013 (3)	0.015 (3)
C15	0.041 (3)	0.060 (3)	0.056 (3)	0.015 (2)	0.011 (2)	0.007 (2)
C210	0.042 (3)	0.064 (3)	0.063 (3)	0.015 (2)	0.014 (2)	0.018 (3)
C214	0.065 (3)	0.044 (3)	0.068 (3)	−0.001 (2)	0.009 (3)	0.011 (2)
C316	0.061 (3)	0.058 (3)	0.054 (3)	0.009 (3)	0.005 (2)	−0.002 (2)
C36	0.036 (2)	0.055 (3)	0.071 (3)	0.009 (2)	0.014 (2)	0.007 (2)
C114	0.103 (5)	0.044 (3)	0.066 (4)	0.020 (3)	0.011 (3)	0.014 (3)
C25	0.047 (3)	0.064 (3)	0.056 (3)	0.018 (2)	0.018 (2)	0.014 (2)
C213	0.052 (3)	0.055 (3)	0.050 (3)	0.007 (2)	0.007 (2)	0.011 (2)
C27	0.069 (4)	0.063 (3)	0.052 (3)	0.027 (3)	−0.001 (3)	0.009 (2)
C37	0.047 (3)	0.049 (3)	0.061 (3)	0.006 (2)	0.019 (2)	0.012 (2)
C315	0.076 (4)	0.038 (3)	0.080 (4)	0.013 (3)	0.019 (3)	0.002 (3)
C216	0.071 (4)	0.055 (3)	0.052 (3)	0.022 (3)	0.006 (3)	0.004 (2)
C314	0.063 (3)	0.042 (3)	0.072 (4)	0.017 (2)	0.020 (3)	0.019 (2)
C16	0.042 (3)	0.066 (3)	0.068 (3)	0.007 (2)	0.020 (2)	0.006 (3)
C28	0.076 (4)	0.045 (3)	0.041 (2)	0.012 (2)	0.009 (2)	0.011 (2)
C215	0.098 (5)	0.043 (3)	0.068 (4)	0.006 (3)	−0.004 (3)	0.003 (3)
C115	0.153 (7)	0.049 (3)	0.065 (4)	0.034 (4)	−0.001 (4)	−0.002 (3)
C116	0.104 (5)	0.051 (3)	0.043 (3)	0.014 (3)	0.010 (3)	0.006 (2)

### Geometric parameters (Å, °)

**Table:**

Cl11—C113	1.737 (5)	C212—H212	0.9299
Cl31—C313	1.730 (5)	C39—C38	1.395 (6)
Cl21—C213	1.728 (5)	C18—C17	1.356 (7)
O12—C11	1.375 (5)	C18—H18	0.9300
O12—C12	1.384 (5)	C310—H310A	0.9700
C34—C35	1.398 (6)	C310—H310B	0.9700
C34—C39	1.398 (6)	C33—H33	0.9300
C34—C33	1.439 (6)	C211—C216	1.374 (7)
O32—C31	1.377 (5)	C211—C210	1.499 (7)
O32—C32	1.378 (5)	C17—C16	1.382 (8)
O22—C21	1.374 (6)	C17—H17	0.9299
O22—C22	1.394 (5)	C38—C37	1.369 (7)
C14—C15	1.395 (6)	C38—H38	0.9300
C14—C19	1.398 (6)	C312—C313	1.377 (6)
C14—C13	1.432 (6)	C312—H312	0.9300
C23—C22	1.311 (6)	C313—C314	1.365 (7)
C23—C24	1.422 (6)	C111—C116	1.366 (7)
C23—H23	0.9299	C26—C25	1.367 (7)
C19—C18	1.394 (6)	C26—C27	1.375 (8)
C19—C11	1.451 (6)	C26—H26	0.9301
C11—O11	1.197 (5)	C15—C16	1.381 (7)
O31—C31	1.199 (5)	C15—H15	0.9300
C31—C39	1.452 (6)	C210—H210A	0.9700
C29—C28	1.396 (7)	C210—H210B	0.9701
C29—C24	1.406 (6)	C214—C213	1.360 (7)
C29—C21	1.440 (7)	C214—C215	1.371 (8)
C35—C36	1.368 (7)	C214—H214	0.9301
C35—H35	0.9300	C316—C315	1.379 (8)
C24—C25	1.391 (7)	C316—H316	0.9300
O21—C21	1.211 (5)	C36—C37	1.378 (7)
C110—C12	1.498 (6)	C36—H36	0.9300
C110—C111	1.506 (6)	C114—C115	1.383 (8)
C110—H110A	0.9700	C114—H114	0.9300
C110—H110B	0.9699	C25—H25	0.9300
C32—C33	1.313 (6)	C27—C28	1.361 (8)
C32—C310	1.503 (6)	C27—H27	0.9299
C311—C312	1.379 (6)	C37—H37	0.9301
C311—C316	1.383 (7)	C315—C314	1.364 (8)
C311—C310	1.502 (6)	C315—H315	0.9299
C13—C12	1.315 (6)	C216—C215	1.383 (8)
C13—H13	0.9301	C216—H216	0.9300
C22—C210	1.500 (7)	C314—H314	0.9300
C113—C114	1.346 (7)	C16—H16	0.9300
C113—C112	1.372 (6)	C28—H28	0.9300
C112—C111	1.375 (7)	C215—H215	0.9301
C112—H112	0.9301	C115—C116	1.379 (8)
C212—C213	1.378 (7)	C115—H115	0.9299
C212—C211	1.388 (7)	C116—H116	0.9301
			
C11—O12—C12	122.6 (3)	C216—C211—C212	119.0 (4)
C35—C34—C39	118.9 (4)	C216—C211—C210	120.8 (5)
C35—C34—C33	123.0 (4)	C212—C211—C210	120.2 (5)
C39—C34—C33	118.0 (4)	C18—C17—C16	120.0 (5)
C31—O32—C32	122.6 (3)	C18—C17—H17	119.9
C21—O22—C22	122.0 (4)	C16—C17—H17	120.1
C15—C14—C19	118.3 (4)	C37—C38—C39	119.5 (4)
C15—C14—C13	122.7 (4)	C37—C38—H38	120.3
C19—C14—C13	119.0 (4)	C39—C38—H38	120.2
C22—C23—C24	121.3 (4)	C313—C312—C311	119.9 (4)
C22—C23—H23	119.3	C313—C312—H312	120.1
C24—C23—H23	119.4	C311—C312—H312	120.0
C18—C19—C14	120.6 (4)	C314—C313—C312	121.6 (5)
C18—C19—C11	120.0 (4)	C314—C313—Cl31	118.7 (4)
C14—C19—C11	119.4 (4)	C312—C313—Cl31	119.7 (4)
O11—C11—O12	116.5 (4)	C116—C111—C112	118.1 (5)
O11—C11—C19	126.5 (4)	C116—C111—C110	120.6 (5)
O12—C11—C19	117.0 (4)	C112—C111—C110	121.4 (4)
O31—C31—O32	116.6 (4)	C25—C26—C27	120.9 (5)
O31—C31—C39	126.7 (4)	C25—C26—H26	119.7
O32—C31—C39	116.6 (3)	C27—C26—H26	119.4
C28—C29—C24	120.2 (4)	C16—C15—C14	120.0 (5)
C28—C29—C21	120.3 (4)	C16—C15—H15	120.0
C24—C29—C21	119.4 (4)	C14—C15—H15	120.0
C36—C35—C34	119.7 (4)	C211—C210—C22	112.5 (4)
C36—C35—H35	120.2	C211—C210—H210A	109.4
C34—C35—H35	120.1	C22—C210—H210A	109.3
C25—C24—C29	118.0 (4)	C211—C210—H210B	108.9
C25—C24—C23	123.3 (4)	C22—C210—H210B	108.8
C29—C24—C23	118.7 (4)	H210A—C210—H210B	107.9
C12—C110—C111	112.5 (4)	C213—C214—C215	118.8 (5)
C12—C110—H110A	108.9	C213—C214—H214	120.7
C111—C110—H110A	109.0	C215—C214—H214	120.5
C12—C110—H110B	109.3	C315—C316—C311	120.4 (5)
C111—C110—H110B	109.3	C315—C316—H316	119.9
H110A—C110—H110B	107.8	C311—C316—H316	119.7
C33—C32—O32	121.5 (4)	C35—C36—C37	121.2 (5)
C33—C32—C310	129.2 (4)	C35—C36—H36	119.3
O32—C32—C310	109.3 (4)	C37—C36—H36	119.5
C312—C311—C316	118.5 (4)	C113—C114—C115	118.1 (5)
C312—C311—C310	120.7 (4)	C113—C114—H114	120.9
C316—C311—C310	120.8 (4)	C115—C114—H114	121.0
C12—C13—C14	120.7 (4)	C26—C25—C24	120.7 (5)
C12—C13—H13	119.6	C26—C25—H25	119.6
C14—C13—H13	119.6	C24—C25—H25	119.7
C23—C22—O22	120.9 (4)	C214—C213—C212	121.9 (5)
C23—C22—C210	129.5 (4)	C214—C213—Cl21	118.3 (4)
O22—C22—C210	109.6 (4)	C212—C213—Cl21	119.8 (4)
O21—C21—O22	115.7 (5)	C28—C27—C26	120.3 (5)
O21—C21—C29	126.7 (5)	C28—C27—H27	119.8
O22—C21—C29	117.6 (4)	C26—C27—H27	120.0
C114—C113—C112	121.4 (5)	C38—C37—C36	120.4 (4)
C114—C113—Cl11	118.4 (4)	C38—C37—H37	119.8
C112—C113—Cl11	120.2 (4)	C36—C37—H37	119.8
C113—C112—C111	121.1 (4)	C314—C315—C316	120.9 (5)
C113—C112—H112	119.5	C314—C315—H315	119.5
C111—C112—H112	119.4	C316—C315—H315	119.6
C213—C212—C211	119.3 (5)	C211—C216—C215	120.5 (5)
C213—C212—H212	120.4	C211—C216—H216	119.7
C211—C212—H212	120.3	C215—C216—H216	119.8
C13—C12—O12	121.1 (4)	C315—C314—C313	118.6 (5)
C13—C12—C110	129.7 (4)	C315—C314—H314	120.9
O12—C12—C110	109.2 (4)	C313—C314—H314	120.4
C38—C39—C34	120.3 (4)	C15—C16—C17	120.8 (5)
C38—C39—C31	119.4 (4)	C15—C16—H16	119.5
C34—C39—C31	120.3 (4)	C17—C16—H16	119.6
C17—C18—C19	120.2 (5)	C27—C28—C29	119.9 (5)
C17—C18—H18	119.8	C27—C28—H28	120.1
C19—C18—H18	120.0	C29—C28—H28	120.0
C311—C310—C32	113.3 (4)	C214—C215—C216	120.5 (5)
C311—C310—H310A	109.2	C214—C215—H215	119.9
C32—C310—H310A	109.0	C216—C215—H215	119.6
C311—C310—H310B	108.8	C116—C115—C114	120.9 (5)
C32—C310—H310B	108.6	C116—C115—H115	119.8
H310A—C310—H310B	107.7	C114—C115—H115	119.4
C32—C33—C34	121.0 (4)	C111—C116—C115	120.5 (5)
C32—C33—H33	119.5	C111—C116—H116	119.9
C34—C33—H33	119.5	C115—C116—H116	119.7

### Hydrogen-bond geometry (Å, °)

**Table:**

*D*—H···*A*	*D*—H	H···*A*	*D*···*A*	*D*—H···*A*
C216—H216···O11	0.93	2.56	3.437 (6)	158.
C116—H116···O21	0.93	2.58	3.396 (6)	147.
C26—H26···O22^i^	0.93	2.45	3.339 (7)	161.
C36—H36···O32^i^	0.93	2.52	3.409 (6)	160.

Symmetry codes: (i) *x*+1, *y*, *z*.
